# Near-Infrared Light-Triggered Bacterial Eradication Using a Nanowire Nanocomposite of Graphene Nanoribbons and Chitosan-Coated Silver Nanoparticles

**DOI:** 10.3389/fchem.2021.767847

**Published:** 2021-10-27

**Authors:** Ming Zhou, Hui-Qi Gan, Guo-Rong Chen, Tony D James, Bin Zhang, Qiang Hu, Fugui Xu, Xi-Le Hu, Xiao-Peng He, Yiyong Mai

**Affiliations:** ^1^ Department of General Surgery, Shanghai Xuhui District Dahua Hospital, Shanghai, China; ^2^ Feringa Nobel Prize Scientist Joint Research Center, Key Laboratory for Advanced Materials and Joint International Research Laboratory of Precision Chemistry and Molecular Engineering, School of Chemistry and Molecular Engineering, East China University of Science and Technology, Shanghai, China; ^3^ Department of Chemistry, University of Bath, Bath, United Kingdom; ^4^ School of Chemistry and Chemical Engineering, Henan Normal University, Xinxiang, China; ^5^ School of Chemistry and Chemical Engineering, Frontiers Science Center for Transformative Molecules, Shanghai Key Laboratory of Electrical Insulation and Thermal Ageing, Shanghai Jiao Tong University, Shanghai, China

**Keywords:** chitosan, silver nanoparticles, graphene nanoribbons, antibiotic, nanocomposite

## Abstract

Bacterial infection is a major threat to human health. However, many antibacterial agents currently used are severely limited due to drug-resistance, and the development of side effects. Herein, we have developed a non-antibiotic nanocomposite consisting of chitosan (ChS) coated silver nanoparticles (AgNPs) and graphene nanoribbon (GNR)-based nanowires for light-triggered eradication of bacteria. The presence of AgNP/ChS significantly enhanced the interactions of the GNR nanowires with *Pseudomonas aeruginosa*, a clinically common Gram-negative bacterium. Which enables the highly effective photothermal eradication of bacteria by GNR upon near-infrared light irradiation. The nanocomposite was shown to be applicable for the light-triggered eradication of bacterial biofilms and the inhibition of bacterial growth on medical patches used for abdominal-wall hernia surgery.

## Introduction

Pathogenic microbes have rapidly spread worldwide, which has resulted in a high infection and mortality rates of hospital patients. As such, novel therapeutic strategies to effectively combat microbial infections are urgently required. Bacterial infections have been responsible for millions of deaths (Kupferschmidt, 2016; Morris et al., 2017) and while the discovery of antibiotics substantially reduced the lethal effect of bacterial infections and secondary complications, the overuse of antibacterial agents means that bacterial infections will become an ever-increasing cause of death. The abuse of antibiotics has led to the emergence of multidrug-resistant bacteria (or “superbugs”) such as the methicillin-resistant *Staphylococcus aureus* (MRSA), *Pseudomonas aeruginosa* (*P. aeruginosa*) and *Acinetobacter baumannii* (*A. baumannii*) (Hu et al., 2015; Adegoke et al., 2016; Siriwardena et al., 2018; Hu et al., 2019). These superbugs pose a serious threat to human health. In addition, antibiotics are prohibited from use during for certain incision surgeries such as abdominal wall hernia surgery (Adegoke et al., 2016). As a consequence, the development of non-antibiotic agents to eradicate bacterial infections has become a popular research topic in both academic and industrial environments (Pérez-Köhler et al., 2015).

During the past decade, a number of inorganic and organic materials have been developed with outstanding antimicrobial efficacies (Díez-Pascual, 2020). Among the various materials developed, those with an inherent antimicrobial property and/or the capacity for bacterial eradication with the aid of light have exhibited promise as alternatives for clinically used antibiotics. A representative example is silver nanoparticles (AgNPs) due to their inherent antibacterial activity through the *in-situ* release of toxic silver ions (You et al., 2012). However, AgNPs tend to aggregate, thus reducing the antibacterial efficiency and results in increasing unwanted side effects to normal tissues. To overcome this problem, AgNPs have been attached to the surface of a variety of functional materials such as metal oxides, graphene oxide and organic polymers, leading to an enhanced bioactivity and biocompatibility; this is largely due to the fact that their controlled alignment on the surface of other materials minimizes the non-specific aggregation of the nanoparticles (Yu et al., 2014; Jose et al., 2019). In addition, low-dimensional materials such as GO, thin-layer molybdenum disulfide and manganese oxide can convert photonic energy to heat, achieving photothermal therapy (PTT) of bacterial infections (Chen et al., 2020).

Previously, we have synthesized structurally well-defined graphene nanoribbons (GNR), which self-assemble into one-dimensional nanowires in aqueous solution and as a new class of PTT agents exhibit excellent photothermal-conversion efficiency (Huang et al., 2018; Niu et al., 2019; Luan et al., 2020; Yu et al., 2020). However, unmodified **GNR** lacks the capability to effectively capture bacterial species.

Chitosan (ChS) is a natural polysaccharide consisting of 1,4-β-linked glucosamine units, which can selectively adhere to the membrane of bacteria through electrostatic interactions (Bilal et al., 2017; Asghar et al., 2020). The introduction of ChS to the surface of AgNPs was shown to improve the controlled release of silver ions in bacterial cells, thus enhancing the antibacterial activity of AgNPs (Wang et al., 2019). As a result, we envision that the self-assembly of ChS-coated AgNPs (AgNP/ChS) with GNR nanowires would produce a hybrid material nanocomposite with enhanced antibacterial activity. We envisioned that 1) the presentation of AgNPs on the surface of GNR nanowires could reduce the aggregation of the nanoparticles, thereby enhancing their interaction with bacterial membranes, and 2) the enhanced interaction would subsequently improve the PTT effect of GNRs for bacterial eradication.

**SCHEME 1:**
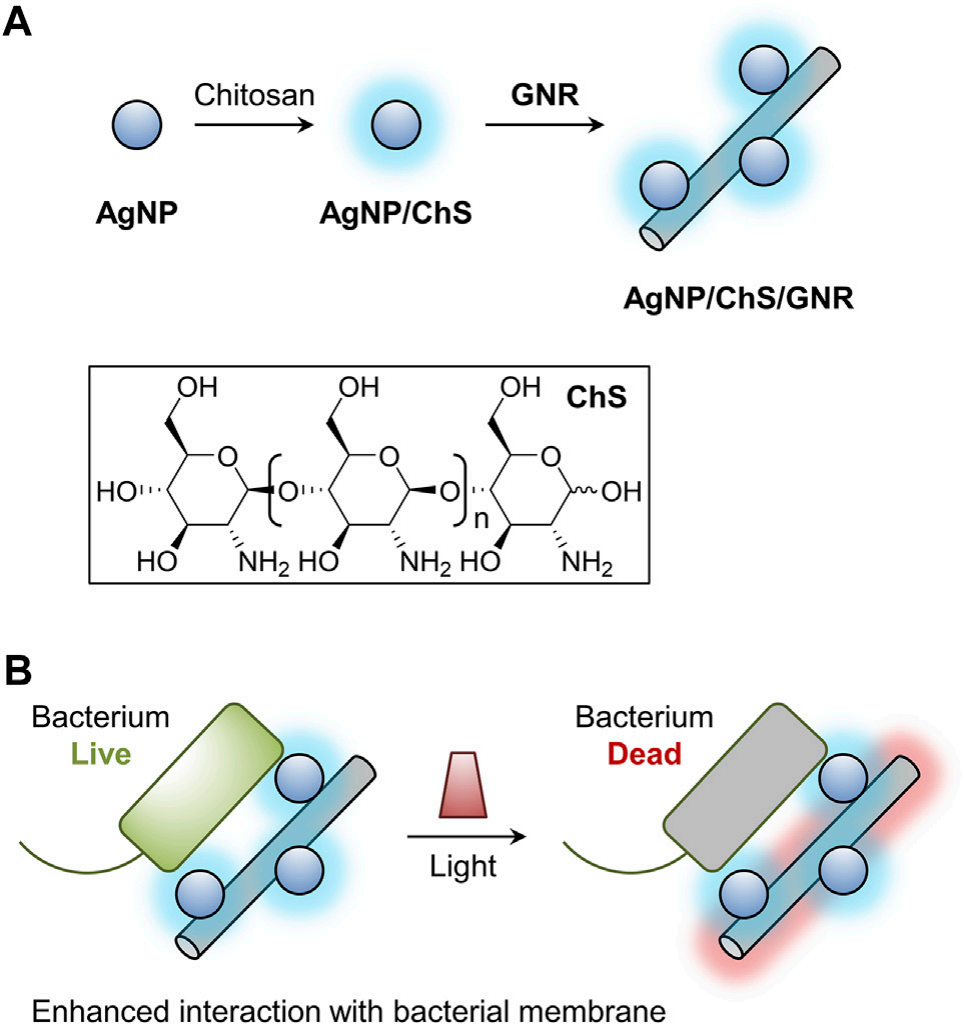
Schematic illustration of **(A)** the construction of the chitosan (ChS)-coated silver nanoparticles (AgNP/ChS) and the material nanocomposite (AgNP/ChS/GNR) formed between AgNP/ChS and granphene nanoribbon (GNR)-based nanowires, and **(B)** light-triggered antibacterial effect of the AgNP/ChS/GNR nanocomposite; the presence of AgNP/ChS enhances the interaction of GNR nanowires with bacterial species, thereby improving the PTT effect of GNRs.

As shown in Scheme 1, chitosan (ChS)-coated AgNPs were prepared, and the resulting AgNP/ChS nanoparticles self-assembled onto GNR-based nanowires, producing a AgNP/ChS/GNR nanocomposite (Scheme 1A). Using a number of techniques, we demonstrated that the presence of AgNP/ChS enhanced the affinity of the nanocomposite for the membrane of a Gram-negative bacterium, thereby enabling the PTT-based eradication of bacterial cells upon light irradiation (Scheme 1B).

## Results and Discussion

**FIGURE 1:**
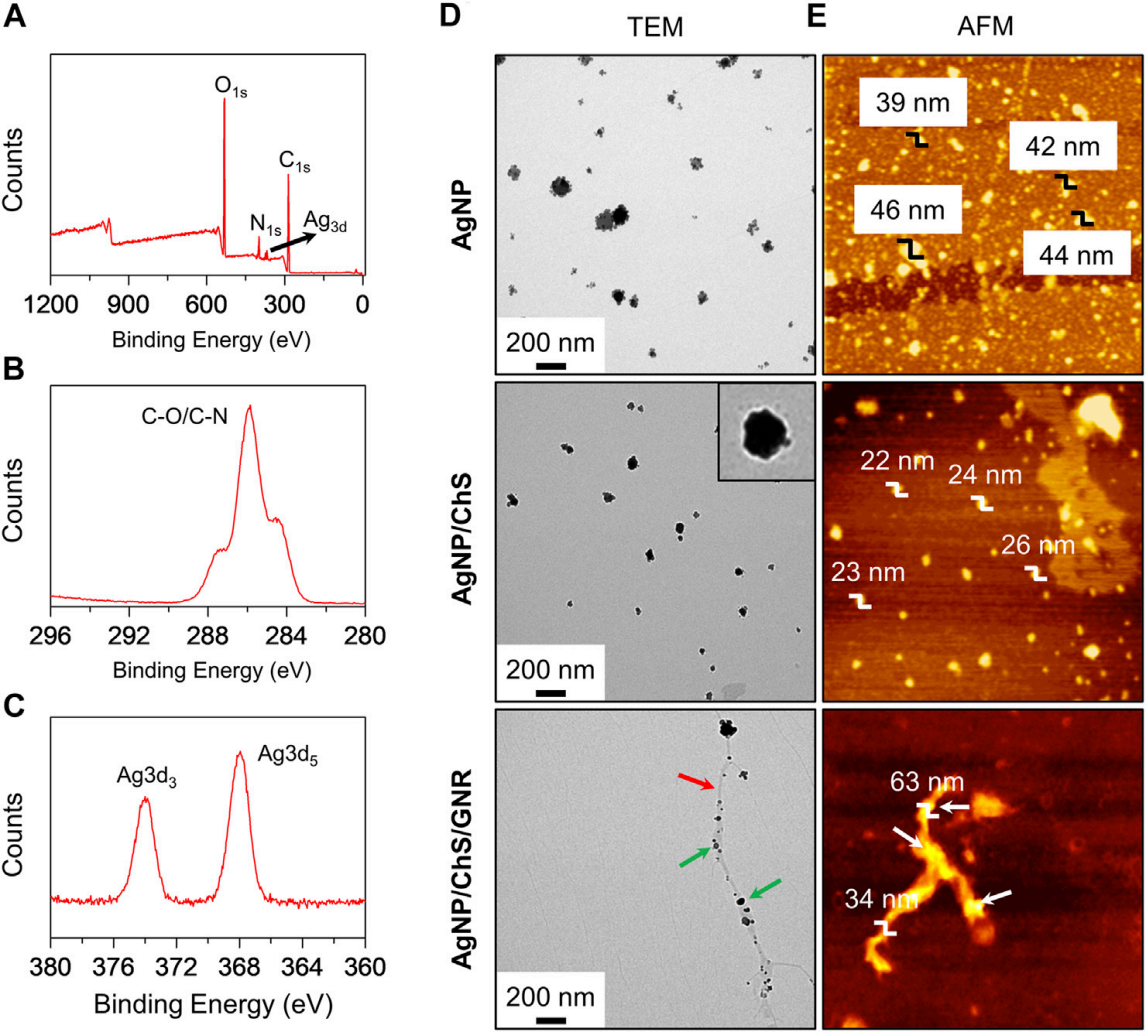
**(A)** Wide-scan XPS spectrum of AgNP/ChS (10 μg ml^−1^/2 mg ml^−1^) corresponding to the characteristic peaks of elemental N, C, O, Ag. **(B)** X-ray photoelectron spectroscopy (XPS) of AgNP/ChS (10 μg ml^−1^/2 mg ml^−1^) corresponding to the characteristic peaks of C-O and C-N. **(C)** XPS of AgNP/ChS (10 μg ml^−1^/2 mg ml^−1^) corresponding to the characteristic peaks of Ag3d. **(D)** Transmission Electron Microscope (TEM) and **(E)** Atom Force Microscope (AFM) images of AgNP (10 μg ml^−1^), AgNP/ChS (10 μg ml^−1^/2 mg ml^−1^), and AgNP/ChS/GNR (10 μg ml^−1^/2 mg ml^−1^/20 μg ml^−1^). The red and green arrows in the TEM image of AgNP/ChS/GNR indicate GNR nanowire and AgNP/ChS, respectively.

For this purpose, ChS with a molecular weight of *ca.* 30 kDa and a deacetylation percentage of 95% was used for the surface modification of AgNPs, producing AgNP/ChS nanoparticles (Arif et al., 2015; Peng et al., 2017). From the UV-vis spectra (Supplementary Figures S1A,C), we observed that the characteristic UV-vis absorption of the AgNPs increased with an increase in the content of ChS in aqueous solution, suggesting that the surface coating of polysaccharides enhanced the aqueous dispersibility of the nanoparticles (Gopinath et al., 2020). X-ray photoelectron spectroscopy (XPS) was then used to characterize the formation of the AgNP/ChS. A wide-scan spectrum established the existence of N, C, O, and Ag elements in the AgNP/ChS system (Figure 1A). Three peaks were observed in the high-resolution C_1s_ spectrum of AgNP/ChS at 287.5, 285.9 and 284.6 eV, which are characteristic signals of the O-C=O, C-N, and C-O peaks of ChS (Carapeto et al., 2017; Xu et al., 2020), respectively (Figure 1B). While in the Ag3d spectrum two characteristic peaks at 373.85 and 367.60 eV, were attributed to the binding energies of Ag 3d_3/2_ and Ag 3d_5/2_ (Kumar-Krishnan et al., 2015; Sun et al., 2020; Xu et al., 2020), respectively (Figure 1C). Significantly, the XPS results indicate the successful construction of AgNP/ChS nanoparticles.

In addition, the morphology of the AgNP and AgNP/ChS nanoparticles were characterized by transmission electron microscopy (TEM) and atomic force microscopy (AFM). We observed morphologically similar nanoparticles from the TEM images of AgNP and AgNP/ChS nanoparticles (Figure 1D); where the former particles (*ca.* 47 nm) were larger than the latter (*ca.* 31 nm). From the representative TEM image of AgNP/ChS, the presence of a polymeric shell on the surface of the AgNP particles was observed, confirming the coating of ChS onto the AgNPs (Figure 1D, inset). In addition, the AFM measurement showed that the height of AgNP particles (39–44 nm) is larger than that of AgNP/ChS (22–26 nm), suggesting that AgNPs with ChS coating are more dispersed.

Next, the self-assembly between AgNP/ChS and GNR was performed. In our previous research, we synthesized a structurally well-defined GNR with covalently grafted polyethylene oxide (PEO) chains on the edges (Huang et al., 2018; Yu et al., 2020). The PEO-modified GNR could aggregate into long nanowires in aqueous solution with an excellent photothermal conversion efficiency of 31%, which surpasses those of other competing low-dimensional materials such as gold nanoparticles, single-walled carbon nanotubes, graphene oxide, thin-layer molybdenum disulfide and manganese oxide under similar experimental conditions (Huang et al., 2018). To prepare a nanocomposite of AgNP/ChS and GNR-based nanowires, AgNP/ChS (20 μg ml^−1^/4 mg ml^−1^) was mixed with GNR (40 μg ml^−1^) in an aqueous solution, followed by sonication (100 W) for 10 min. From the representative UV-vis spectra of the mixture, an increase in the concentration of GNR led to a gradual decrease in the UV-vis absorption of the AgNP/ChS nanoparticles (Supplementary Figures S1B,D). Which indicated that the AgNP/ChS nanoparticles were aligned to the surfaces of the GNR nanowires. In the representative TEM image of the AgNP/ChS/GNR nanocomposite particle-like species were observed to be coated on the surface of wire-like structures of the GNR (Figure 1D). From representative AFM images of the nanocomposite, we observed that the region where the AgNP/ChS nanoparticles are attached, an increased height of 63 nm with respect to that of pure GNR (mean diameter: 34 nm) was observed (Figure 1E). These data confirm the successful formation of a AgNP/ChS/GNR nanocomposite.

**FIGURE 2:**
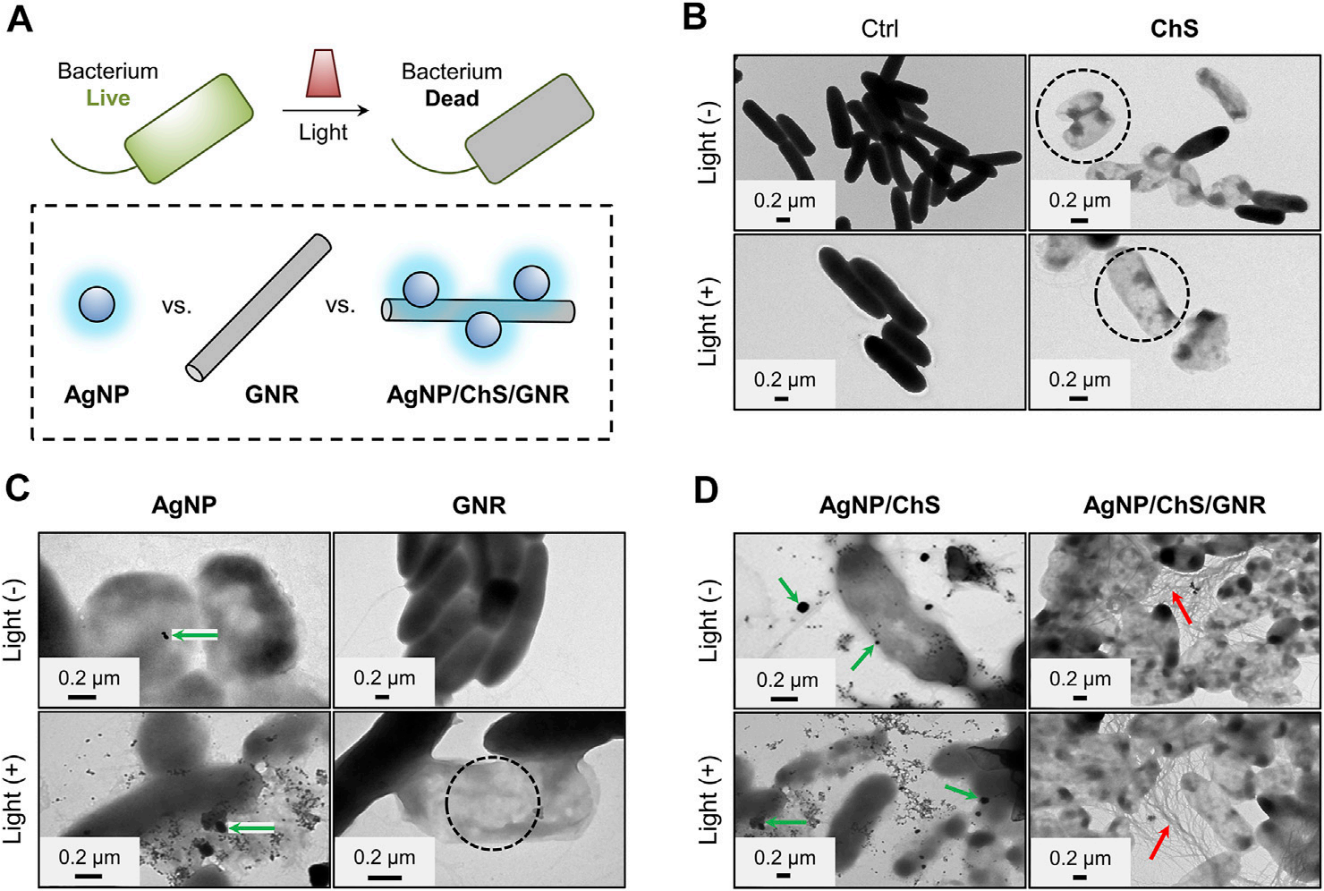
**(A)** Schematic illustration of the light-triggered bacterial eradication using different materials. TEM images of *Pseudomonas aeruginosa* (ATCC 27853, 10^6^ CFU ml^−1^) before and after treatment with **(B)** ChS (2 mg ml^−1^), **(C)** AgNP (10 μg ml^−1^) and GNR (20 μg ml^−1^) **(D)** ChS/AgNP (2 mg ml^−1^/10 μg ml^−1^) and AgNP/ChS/GNR (10 μg ml^−1^/2 mg ml^−1^/20 μg ml^−1^) with and without light irradiation (808 nm, 1 W cm^−2^).

With the nanocomposite in hand, we then evaluated their antibacterial activities under near-infrared (NIR) irradiation (808 nm, 1 W cm^−2^). By optimizing the concentrations of AgNP, ChS and GNR in the composite, we found that AgNP/ChS/GNR with a concentration of 10 μg ml^−1^/2 mg ml^−1^/20 μg ml^−1^ exhibited the best antibacterial efficiency (Supplementary Figure S2). Compared with previously reported AgNP-based antibacterial hybrid materials including ChS/AgNP (35 mg ml^−1^/1 mg ml^−1^) nanocomposites (Pérez-Díaz et al., 2016), ChS/AgNP (9 mg ml^−1^/0.4 mg ml^−1^) nanoparticles within hydrogels (Verma et al., 2017), CNT/Ag (0.9 mg ml^−1^/0.8 mg ml^−1^) nanofiber composites (Jatoi et al., 2020) and ChS hydrogel/AgNP (80 mg ml^−1^/3 mg ml^−1^) nanoparticles (Xie et al., 2018), the concentrations of AgNP and ChS used in the present study were much lower. TEM was used for the visualization of the morphological changes of *Pseudomonas aeruginosa* (ATCC 27853, 10^6^ CFU ml^−1^) in the presence of the AgNP/ChS/GNR nanocomposite; while, AgNP, ChS, AgNP/ChS and GNR were used as controls (Figure 2). Considering the outstanding photothermal property of GNR (Huang et al., 2018; Mousavi et al., 2019; Johnson et al., 2020; Yu et al., 2020; Dou et al., 2021), near-infrared (NIR) irradiation (808 nm, 1 W cm^−2^) was used to evaluate the PTT effect of the materials (Figure 2A). Initially, we observed rod-like morphology in the TEM images of *P. aeruginosa*. The morphology of the bacteria did not change after 808 nm light irradiation in the absence of the materials (Figure 2B), indicating that the NIR light is not harmful to the bacteria. However, in the presence of ChS morphological changes of *P. aeruginosa* (Figure 2B) were observed. Hollow bacterial cells (dead cells) appeared (the black-circled region in the ChS/Light (-)/(+) panels of Figure 2B), which suggests the destruction of the bacteria and the leakage of intracellular components. Similarly, ChS, and AgNPs induced morphological change of the bacteria (from solid to hollow). This was due to the attachment of AgNPs to the membrane of the bacteria as evidenced in the AgNP/Light (-)/(+) panels in Figure 2C). In comparison, the presence of only GNR without light irradiation caused minimal morphological change to the bacteria (Figure 2C), suggesting a minimal antibacterial effect of just GNR. Interestingly, when NIR light irradiation was applied after incubation of GNR with the bacteria, (808 nm, 1 W cm^−2^; temperature increased to ∼51°C) hollow bacterial cells appeared in the TEM (the black-circled region in the GNR/Light (+) panel in Figure 2C). Suggesting that the PTT effect of GNR can result in the death of bacteria.

Subsequently, particle-like species attached to the surface of *P. aeruginosa* were observed in the TEM images of the AgNP/ChS group, (green-arrows in the AgNP/ChS/Light (-)/(+) panels of Figure 2D), while an extensive amount of wire-like species were seen in those of the AgNP/ChS/GNR group (red-arrows in the AgNP/ChS/GNR/Light (-)/(+) panels of Figure 2D). This suggests that, compared to the use of just GNR (bottom-right panel in Figure 2C), the presence of AgNP/ChS enhanced the adhesion of the resulting nanocomposite to the bacterial membrane of (top- and bottom-right panels of Figure 2D). Subsequent light irradiation, resulted in the production of a substantial number of dead bacteria, which was due to the strong PTT effect of the GNRs adjacent to the bacterial cells.

**FIGURE 3:**
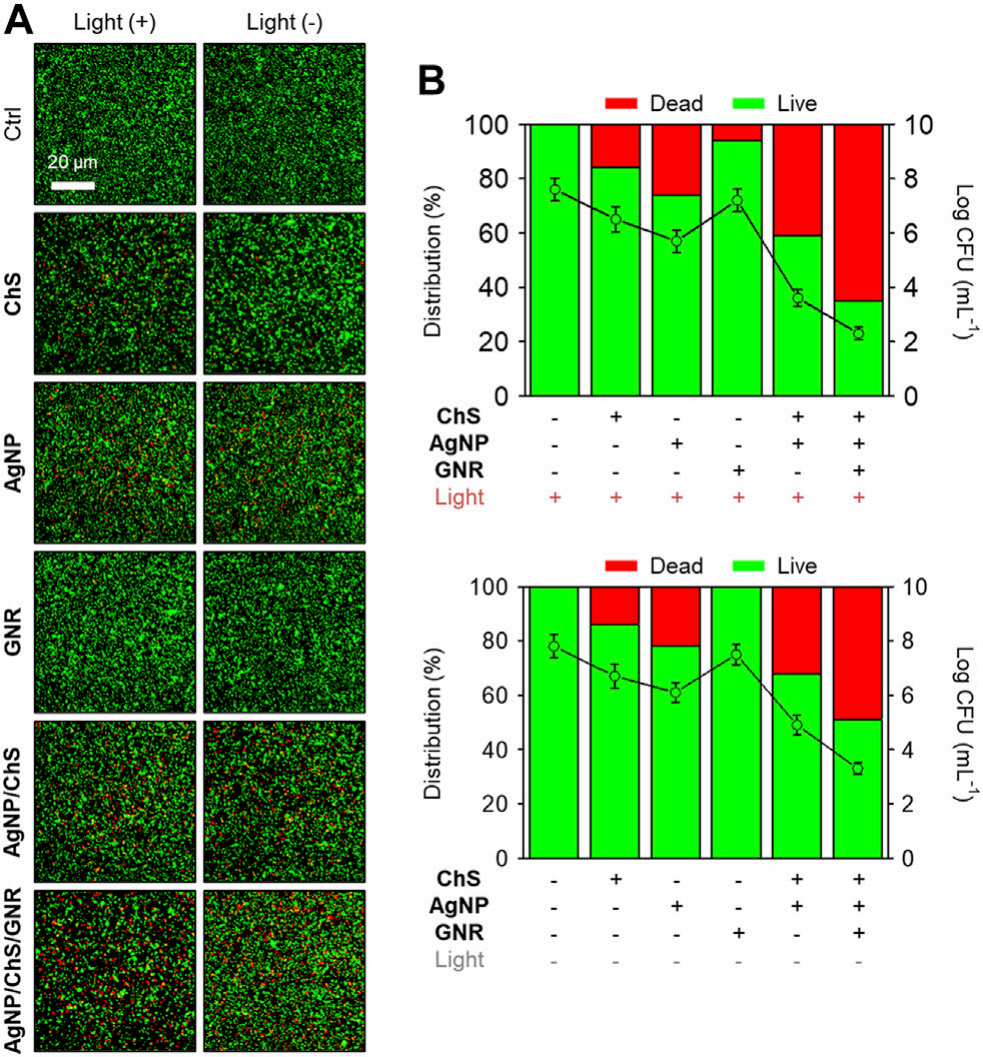
**(A)** Fluorescence imaging and **(B)** quantification of the biofilm formed by *Pseudomonas aeruginosa* (ATCC 27853, 10^6^ CFU ml^−1^) before and after treatment with ChS (2 mg ml^−1^), AgNP (10 μg ml^−1^), GNR (20 μg ml^−1^), ChS/AgNP (2 mg ml^−1^/10 μg ml^−1^) and AgNP/ChS/GNR (10 μg ml^−1^/2 mg ml^−1^/20 μg ml^−1^) with and without light irradiation (808 nm, 1 W cm^−2^). The live and dead cells were stained by Syto9 (1.4 μM, λex/λem = 488/500 nm) and PI (8.3 μM, *λ*ex/*λ*em = 561/600 nm), respectively.

We then evaluated the ability of the AgNP/ChS/GNR nanocomposite to supress *P. aeruginosa*-based biofilm formation (Mathias et al., 2010). After treatment of the biofilms with AgNP, ChS, GNR, AgNP/ChS or AgNP/ChS/GNR in the absence and presence of light irradiation, a live-dead cell staining assay (Mathias et al., 2010; Ou et al., 2019; Ma et al., 2020; Wu et al., 2015) was used to quantitatively determine the antibacterial efficiency (Figure 3). Compared to the blank group, the presence of just ChS or AgNPs resulted in minimal bacterial death in the biofilms, irrespective of light irradiation (Figures 3A,B). In addition, AgNP/ChS (32% dead cells produced) exhibited a better antibacterial activity than AgNPs (22% dead cells produced) or ChS (14% dead cells produced) alone, which agrees with previous observations that the presence of ChS can enhance the antimicrobial activity of AgNPs (Kumar-Krishnan et al., 2015).

The use of GNR without light irradiation hardly killed any bacteria, and NIR light slightly increased the antibacterial activity of the GNR (Figures 3A,B). These observations confirm the weak association between unmodified GNR and the bacteria. Interestingly, we observed that the AgNP/ChS/GNR nanocomposite exhibited the best antibacterial effect amongst all the groups. The enhanced activity of the nanocomposite (49% dead cells produced) than that of AgNP/ChS (32% dead cells produced) was attributed to an increase in the active surface area of the nanoparticles when aligned on the GNR nanowires. Subsequent NIR irradiation (808 nm, 1 W cm^−2^) increased the percentage of dead cells in the biofilm to 65%. This observation confirmed that the presence of AgNP/ChS significantly enhances the interaction of the GNR with bacteria, thereby facilitating the PTT-based eradication of bacterial cells in the biofilm.

**FIGURE 4:**
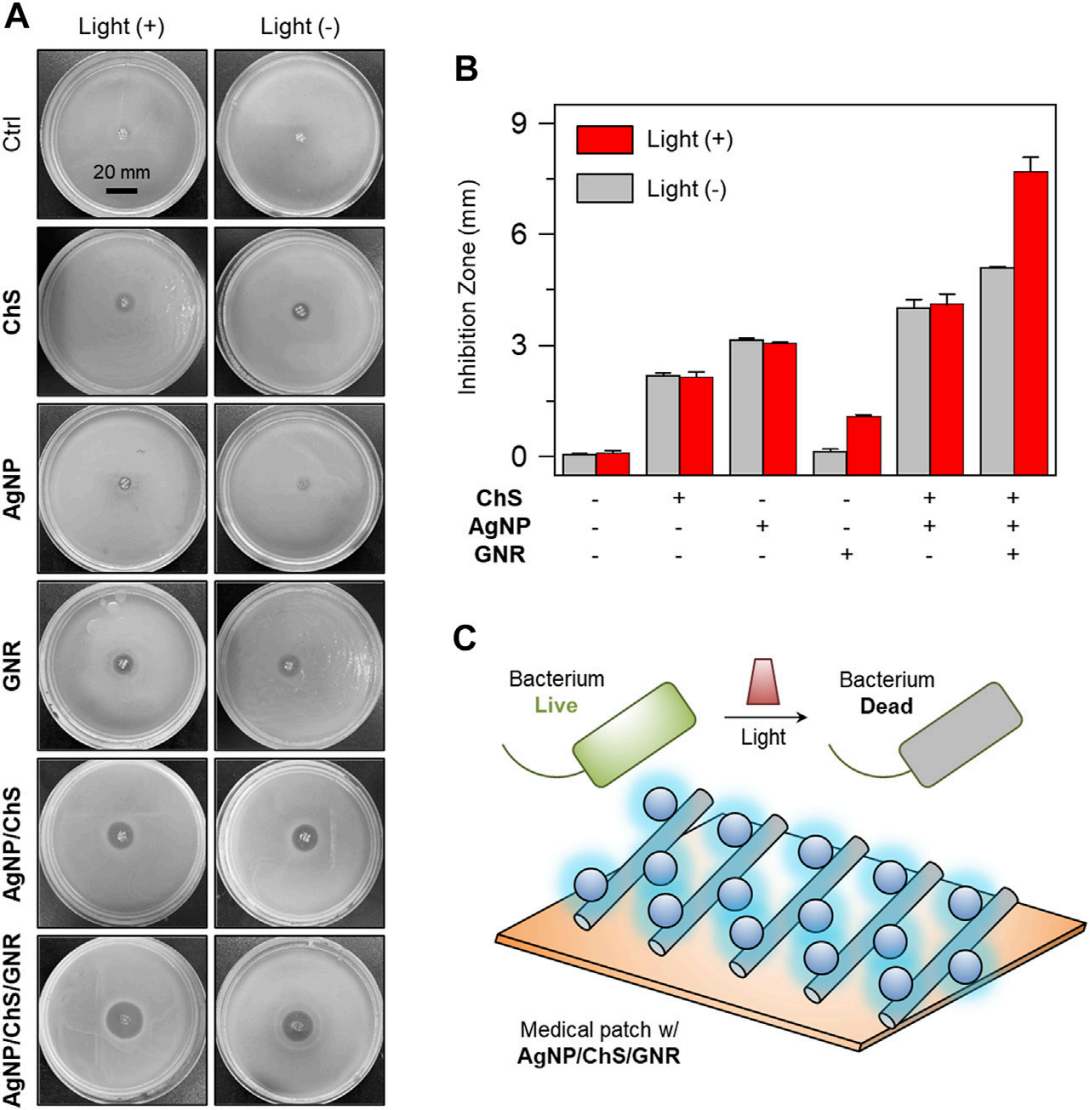
**(A)** Photos (taken by a Nikon camera) and **(B)** diameters of the inhibition zone produced on Luria–Bertani (LB) agar plates cultured with *Pseudomonas aeruginosa* (ATCC 27853, 106 CFU ml^−1^) after treatment with ChS (2 mg ml^−1^), AgNP (10 μg ml^−1^), GNR (20 μg ml^−1^), ChS/AgNP (2 mg ml^−1^/10 μg ml^−1^) and AgNP/ChS/GNR (10 μg ml^−1^/2 mg ml^−1^/20 μg ml^−1^) with and without light irradiation (808 nm, 1 W cm^−2^). **(C)** Schematic illustration of the light-triggered bacterial eradication using AgNP/ChS/GNR on medical patch.

To demonstrate the potential clinical applicability of the nanocomposite, AgNP/ChS/GNR as well as the control materials AgNP, ChS, GNR and AgNP/ChS were used to coat a medical patch made of monofilament polypropylene (PP). Medical patches are clinically used for the treatment of extra-abdominal hernia. However, the application of the synthetic patches can result in bacterial infection, leading to secondary complications. Therefore, we coated the patches with different antibacterial materials, and then the modified patches were placed in the middle of an agar culture plate covered by *P. aeruginosa*. After 24 h, the diameters of the inhibition zone produced by the modified patches were measured (Figure 4) (Wei et al., 2019). Compared to the blank group, the ChS and AgNP coated patches produced an inhibition zone of 2 and 3 mm, respectively (Figures 4A,B); the size of the inhibition zone was independent of light irradiation. The use of NIR light slightly increased the inhibition zone of the **GNR**-coated patch from 0 to 1 mm, suggesting a low level of interaction between the unmodified nanowires and the bacteria. However, in sharp contrast, the AgNP/ChS/GNR coated patch produced an inhibition zone of 5 mm, and subsequent light irradiation increased the diameter further to 7 mm. This finding confirms that AgNP/ChS on the surface of the nanocomposite enhanced the interaction between the GNR and bacteria, enabling light-triggered eradication (PTT) of the bacteria.

## Conclusion

In summary, we have constructed a nanocomposite formed between ChS-coated AgNPs and GNR-based nanowires. Microscopic analyses confirmed the attachment of the AgNP/ChS particles onto the surface of the GNR nanowires, indicating that supramolecular self-assembly between the two nanomaterials had successfully occurred. To the best of our knowledge, this is the first example reporting the construction of a nanocomposite between structurally well-defined GNR and inorganic nanoparticles, thereby offering scope for the development of other material-based composites for biomedical applications (Dou et al., 2019). The biological assays indicate that AgNP/ChS particles on the surface of GNR significantly enhanced the interaction of the resulting nanocomposite with *P. aeruginosa*, and enabled effective NIR light-triggered bacterial eradication due to the outstanding PTT effect of water-soluble GNR (Huang et al., 2018; Yu et al., 2020) More interestingly, we demonstrated the effective use of the nanocomposite, for coating medical patches, for the suppression of bacterial growth on and the effect could be enhanced by NIR light irradiation. Our research illustrates the potential of developing antibacterial patches that can be used to overcome unwanted bacterial infections following abdominal-wall hernia surgery.

## Data Availability

The original contributions presented in the study are included in the article/Supplementary Material, further inquiries can be directed to the corresponding authors.
