# Supplementary material for: Near-Infrared Light-Triggered Bacterial Eradication Using a Nanowire Nanocomposite of Graphene Nanoribbons and Chitosan-Coated Silver Nanoparticles
Source: Front Chem. 2021 Oct 27;9:767847. doi: 10.3389/fchem.2021.767847 (PMC8579076; doi:10.3389/fchem.2021.767847)
Supplement: Supplementary file 1 [file DataSheet1.docx]

Supplementary Material

# Contents list

2. Experimental Section

3. Additional Figures

4. Additional References

# Experimental Section

All chemicals and reagents purchased are of analytical grade. Argentum nitricum (AgNO_3_) and chitosan (**ChS**) were purchased from Meryer Technologies Co., Ltd. Acetic acid (100%) and sodium citrate were purchased from Etiqueta (CE). Nutrient agar was purchased from Beyotime Biotechnology. The UV-vis absorption spectra were measured on a Varian Cary 500 UV-vis spectrophotometer. Elemental analysis was measured on an X-ray photoelectron spectrometer. Fluorescence imaging of bacteria was carried out using a Nikon AIR Confocal Laser-Scanning Microscope. The standard-type of *Pseudomonas aeruginosa* (*P. aeruginosa*) used in this study (ATCC 27853) was obtained from Beijing Zhongyuan Ltd.

**Synthesis of silver nanoparticles (AgNPs) and AgNP/ChS. AgNP**s were prepared by the reduction of silver nitrate with sodium citrate according to previous methods (Tripathi et al., 2011). An aqueous solution of silver nitrate (10 mmol L^-1^, 500 mL) was heated to 90 °C, and then stirred gently with a magnetic stirrer. Then, a preheated sodium citrate solution (3 mol L^-1^, 5.0 mL) was added to the solution at 90 °C. When the color of the solution turned yellow, with the solvent was removed rotary evaporation, followed by oven drying. After drying, fine-black particles were obtained, and were re-dispersed in distilled water. The as-prepared **AgNP**s solution (100 μL) was added into a chitosan solution (2 mg mL^-1^, 10 mL), and the mixture was stirred for 2 hours to obtain **AgNP/ChS** (Arif et al., 2015).

**Preparation of AgNP/ChS/GNR. AgNP/ChS** (1 mL, 20 μg mL^-1^/4 mg mL^-1^) was sonicated (100 W) for 10 min to obtain a well-dispersed **AgNP/ChS** solution. Then, this dispersion was added to a Tris-HCl (0.01 M, pH 7.4) solution of **GNR** (1 mL, 0.4 mg mL^-1^) prepared according to our previously reported procedure (Yu et al., 2020). The resulting mixture was sonicated (100 W) for 10 min to obtain the **AgNP/ChS/GNR** nanocomposite.

**High-resolution transmission electron microscopy (HR-TEM).** A droplet of **AgNP** (10 μg mL^-1^), **AgNP/ChS** (10 μg mL^-1^/2 mg mL^-1^) or **AgNP/ChS/GNR** (10 μg mL^-1^/2 mg mL^-1^/20 μg mL^-1^) was dropped onto 200-mesh holey carbon copper grids. Then, images were recorded with JEOL 2100 equipped with a Gatan Orius charged-coupled device camera and Tridiem energy filter operating at 200 kV.

**Atomic force microscopy (AFM).** AFM (AJ-III, Aijian nanotechnology Inc., China) images of the materials were recorded in the tapping mode to simultaneously collect height and phase data. For sample preparation, 100 μL Tris-HCl (0.01 M, pH 7.4) solution of **AgNP**s (10 μg mL^-1^), **AgNP/ChS** (10 μg mL^-1^/2 mg mL^-1^) or **AgNP/ChS/GNR** (10 μg mL^-1^/2 mg mL^-1^/20 μg mL^-1^) were cast onto a freshly cleaved mica surface, followed by drying at room temperature prior to analysis.

**Measurement of the photothermal effect of materials in solution.** The photothermal properties of **GNR** (20 μg mL^-1^) was determined by measuring the temperature elevation induced by NIR laser irradiation (808 nm, 1 W cm^-2^). Typically, sample was placed in an EP tube and irradiated with NIR light (808 nm; 1 W cm^-2^) for 5 min. The laser-induced temperature elevation was recorded using an IR camera (IR Tech Co. Ltd., Shanghai, China).

**Antibacterial activity of the materials measured by the disc diffusion method.** The antibacterial activity of medical patches coated with **AgNP** (10 μg mL^-1^), **ChS** (2 mg mL^-1^), **GNR** (20 μg mL^-1^), **AgNP/ChS** (10 μg mL^-1^/2 mg mL^-1^) and **AgNP/ChS/GNR** (10 μg mL^-1^/2 mg mL^-1^/20 μg mL^-1^) was tested using the modified agar diffusion assay (disc test) against *Pseudomonas aeruginosa* with or without 808 nm (1 W cm^-2^, 20 min) irradiation. Bacteria were cultured in the presence of the patches on the discs at 37 °C for 24 h. Then, the diameter of the inhibition zone produced around the patches was measured.

**Analysis of bacterial morphology by HR-TEM.** *P. aeruginosa* (ATCC 27853) was grown in LB (Luria-Bertani) medium to OD_600_ = 0.1, and then incubated with **AgNP** (10 μg mL^-1^), **ChS** (2 mg mL^-1^), **GNR** (20 μg mL^-1^), **AgNP/ChS** (10 μg mL^-1^/2 mg mL^-1^) or **AgNP/ChS/GNR** (10 μg mL^-1^/2 mg mL^-1^/20 μg mL^-1^). Then, bacteria were treated with NIR light (808 nm, 1 W cm^-2^) for 20 min. After incubation for 2 h, the resulting bacteria were collected and fixed in 2.5% glutaraldehyde (in phosphate buffered saline (PBS)) over night, and then washed with PBS twice after fixation. Then, bacteria were dehydrated using an ethanol series with an increasing concentration (30%, 40%, 50%, 60%, 70%, 80%, 90%, pure ethanol) for 15 min, and finally suspended in pure *t*-butanol for 15 min. Bacteria were air-dried and coated with gold for TEM imaging on a Hitachi S4800 Transmission Electron Microscope.

**Eradication of *Pseudomonas aeruginosa*-based biofilms.** *P. aeruginosa* (ATCC 27853) from a single colony was cultured in the LB medium (2 mL). Then, the culture was placed in an orbital shaker (180 r. p. m.) for 16 h at 37 °C. The resulting culture was diluted with fresh LB medium to an OD_600_ of 0.02, and then was moved to a 96-well microtiter plate. The resulting cultures were incubated for 16 h at 37 °C to obtain the *P. aeruginosa*-based biofilms (Mathias et al., 2010). To evaluate antibacterial activity, the established *P. aeruginosa* biofilms were coated on glass slides, followed by addition of sterile water (100 μL; control), **ChS** (100 μL, 2 mg mL^-1^), **AgNP** (100 μL, 10 μg mL^-1^), **GNR** (100 μL, 20 μg mL^-1^), **AgNP/ChS** (100 μL, 10 μg mL^-1^/2 mg mL^-1^/) or **AgNP/ChS/GNR** (100 μL, 10 μg mL^-1^/2 mg mL^-1^/20 μg mL^-1^). Then, the glass slides were irradiated with 808 nm laser light (1 W cm^-2^) for 20 min. Each glass slide was then incubated for 16 h at 37 °C. Then, 20 μL of live/dead staining solution (Syto9/PI) was added onto the glass slices with a final concentration of Syto9 and PI being 1.4 μM and 8.3 μM, respectively. Then, the glass slides were placed inside a well-plate covered with a fresh air-permeable foil, and then moved to an incubator for 2 h at 37 °C before imaging. The different proportions of green (Syto9: live bacteria) and red (PI: dead bacteria) biovolumes were calculated by analyzed imaging stacks.

# Additional Figures


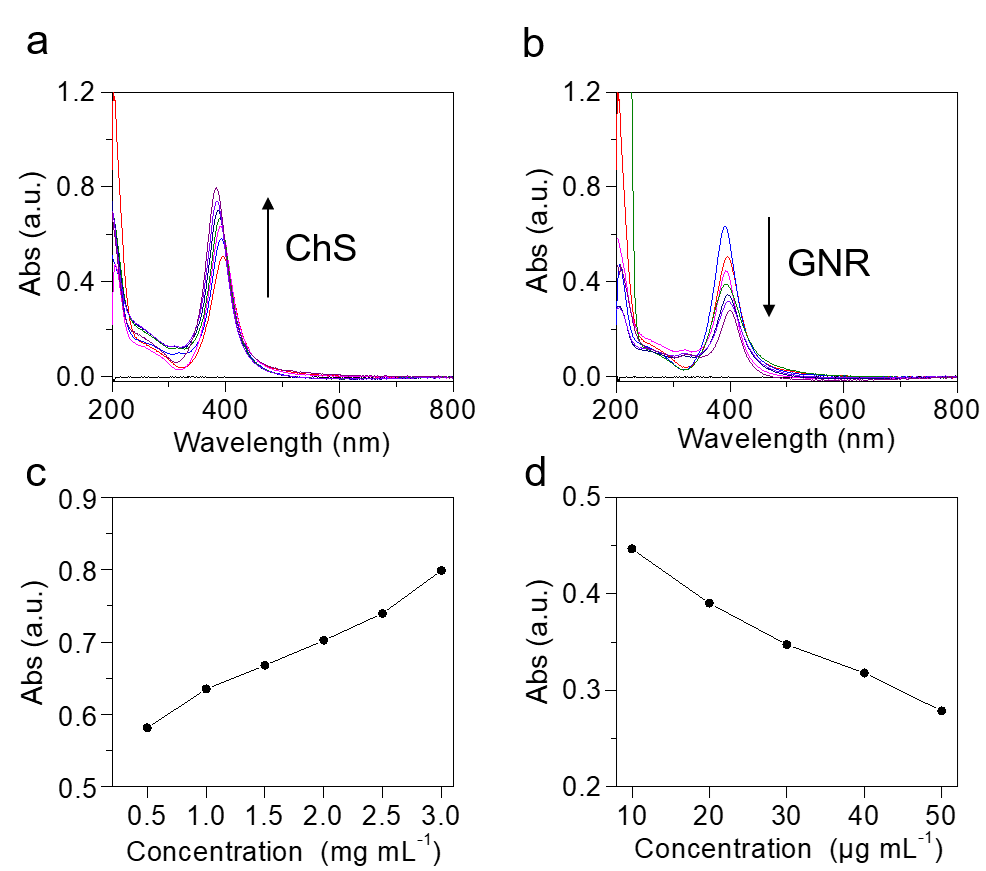


**Figure S1.** UV-vis absorption spectra of (a) **AgNP** (10 μg mL^-1^) with increasing **ChS** (from bottom to top curve: 0.5, 1.0, 1.5, 2.0, 2.5 and 3.0 mg mL^-1^) in Tris-HCl (0.01 M, pH 7.4), and (b) **AgNP/ChS** (10 μg mL^-1^/2 mg mL^-1^) in Tris-HCl (0.01 M, pH 7.4) with increasing **GNR** (from top to bottom curve: 10, 20, 30, 40 and 50 μg mL^-1^). (c) Plotting the maximum UV-vis absorption intensity of **AgNP** as a function of **ChS** concentration. (d) Plotting the maximum UV-vis absorption intensity of **AgNP/ChS** as a function of **GNR** concentration.

**
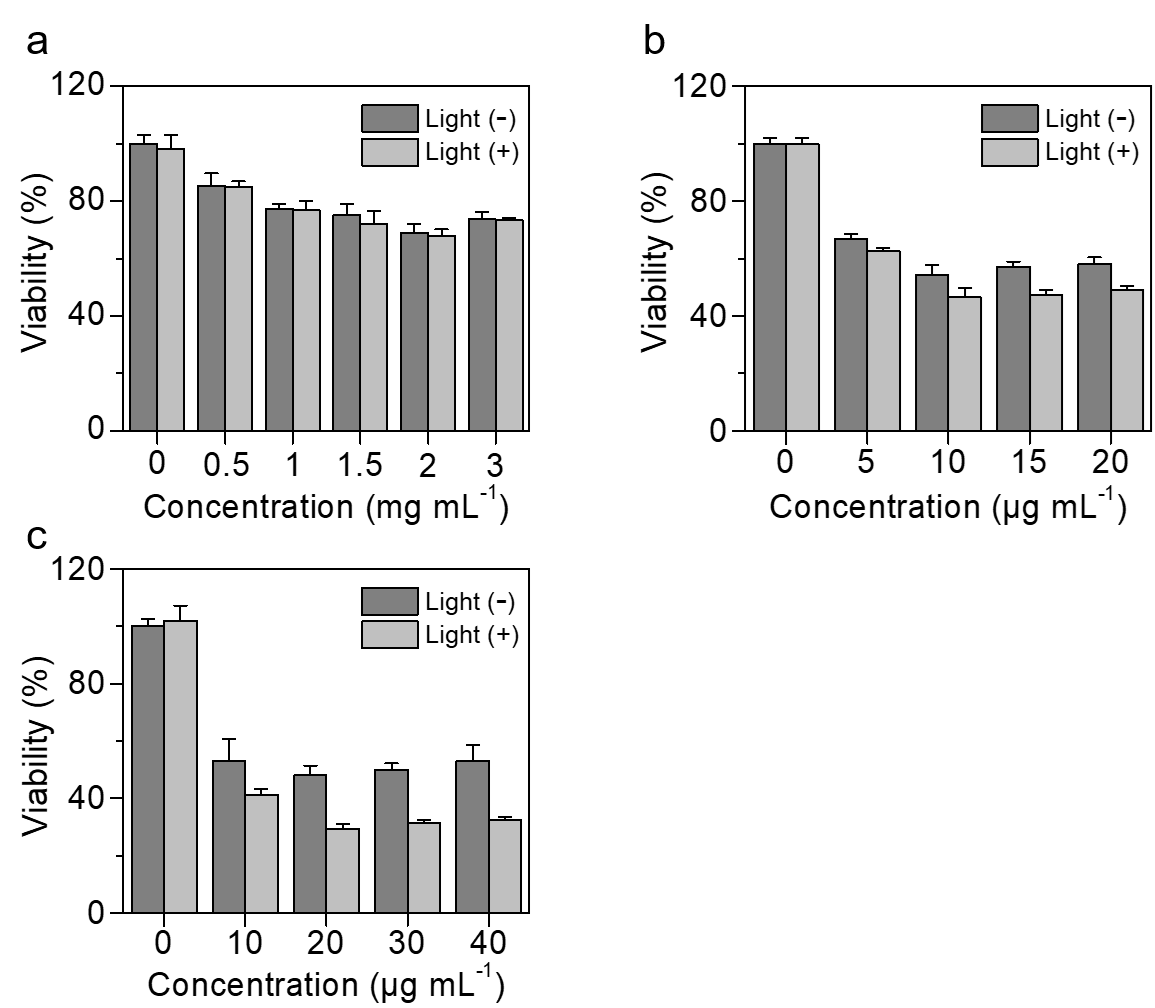
**

**Fig. S2.** (a) Viability of *P. aeruginosa* (ATCC 27853, 10^6^ CFU mL^-1^) in the presence of increasing **ChS** without and with light irradiation (808 nm, 1 W cm^-2^ for 20 min). (b) Viability of *P. aeruginosa* (ATCC 27853, 10^6^ CFU mL^-1^) in the presence of **ChS** (2 mg mL^-1^) and increasing **AgNP** without and with light irradiation (808 nm, 1 W cm^-2^ for 20 min). (c) Viability of *P. aeruginosa* (ATCC 27853, 10^6^ CFU mL^-1^) in the presence of **AgNP/ChS** (10 μg mL^-1^/2 mg mL^-1^) and increasing **GNR** without and with light irradiation (808 nm, 1 W cm^-2^ for 20 min).

# Additional References

Arif, D., Niazi, M. B. K., Ul-Haq, N., Anwar, M. N., Hashmi, E. (2015). Preparation of Antibacterial Cotton Fabric Using Chitosan-silver Nanoparticles. *Fiber. Polym*. 16, 1519-1526. doi: 10.1007/s12221-015-5245-6

Mathias, M., Stefano, D. -F., Ute, R., Susanne, H. (2010). A 96-well-plate–based optical method for the quantitative and qualitative evaluation of Pseudomonas aeruginosa biofilm formation and its application to susceptibility testing. *Nat. Protoc*. 5, 1460-1469. doi: 10.1038/nprot.2010.110

Yu, Z.-H., Li, X.-S., Xu, F.-G., Hu, X.-L., Yan, J.-T., Kwon, N., Chen, G.-R., Tang, T.-T., Dong, X.-J., Mai, Y.-Y., Chen, D.-J., Yoon, J., He, X.-P., Tian, H. (2020). A Supramolecular-Based Dual-Wavelength Phototherapeutic Agent with Broad-Spectrum Antimicrobial Activity Against Drug-Resistant Bacteria. *Angew. Chem. Int. Ed*. 59, 3658-3664. doi: 10.1002/anie.201913506

Tripathi, S., Mehrotra, G. K., Dutta, P. K. (2011). Chitosan–silver oxide nanocomposite film: Preparation and antimicrobial activity. *Bull. Mater. Sci*. 34, 29-35. doi: 10.1007/s12034-011-0032-5
